# First description of *Candida nivariensis* in Brazil: antifungal susceptibility profile and potential virulence attributes

**DOI:** 10.1590/0074-02760150376

**Published:** 2016-01

**Authors:** Maria Helena Galdino Figueiredo-Carvalho, Livia de Souza Ramos, Leonardo Silva Barbedo, Alessandra Leal da Silva Chaves, Ilda Akemi Muramoto, André Luis Souza dos Santos, Rodrigo Almeida-Paes, Rosely Maria Zancopé-Oliveira

**Affiliations:** 1 Fundação Oswaldo Cruz, Instituto Nacional de Infectologia Evandro Chagas, Laboratório de Micologia, Rio de Janeiro, RJ, Brasil; 2 Universidade Federal do Rio de Janeiro, Instituto de Microbiologia Paulo de Góes, Departamento de Microbiologia Geral, Laboratório de Investigação de Peptidases, Rio de Janeiro, RJ, Brasil; 3 Instituto Nacional de Câncer, Laboratório de Micologia, Rio de Janeiro, RJ, Brasil

**Keywords:** Candida nivariensis, Brazil, antifungal resistance, hydrolytic enzymes, adherence, biofilm

## Abstract

This study evaluated the antifungal susceptibility profile and the production of potential virulence attributes in a clinical strain of *Candida nivariensis* for the first time in Brazil, as identified by sequencing the internal transcribed spacer (ITS)1-5.8S-ITS2 region and D1/D2 domains of the 28S of the rDNA. For comparative purposes, tests were also performed with reference strains. All strains presented low planktonic minimal inhibitory concentrations (PMICs) to amphotericin B (AMB), caspofungin (CAS), and voriconazole. However, our strain showed elevated planktonic MICs to posaconazole (POS) and itraconazole, in addition to fluconazole resistance. Adherence to inert surfaces was conducted onto glass and polystyrene. The biofilm formation and antifungal susceptibility on biofilm-growing cells were evaluated by crystal violet staining and a XTT reduction assay. All fungal strains were able to bind both tested surfaces and form biofilm, with a binding preference to polystyrene (p < 0.001). AMB promoted significant reductions (≈50%) in biofilm production by our *C. nivariensis* strain using both methodologies. This reduction was also observed for CAS and POS, but only in the XTT assay. All strains were excellent protease producers and moderate phytase producers, but lipases were not detected. This study reinforces the pathogenic potential of *C. nivariensis* and its possible resistance profile to the azolic drugs generally used for candidiasis management.

*Candida glabrata* is an emerging pathogen in public and private Brazilian hospitals (Colombo et al. 2013). Moreover, it is the most common species of invasive fungal infections among non-albicans *Candida*species in North America (Lockhart et al. 2012, Pfaller et al. 2014) and Central Europe (De Luca et al. 2012, Milazzo et al. 2014). Based on molecular analysis, two new species that are closely related to and phenotypically resemble *C. glabrata* have been described: *Candida nivariensis* and*Candida bracarensis* (Alcoba-Flórez et al. 2005b, Correia et al. 2006). *C. nivariensis*was first described in 2005 after it was isolated from clinical samples (bronchoalveolar lavage, blood culture, and urine) from three patients in the Canary Islands, which are African islands under Spanish rule (Alcoba-Flórez et al. 2005b). MALDI-TOF analyses were later demonstrated to be an efficient tool to differentiate these species (Gorton et al. 2013). Since then, other cases have been reported in Europe (Borman et al. 2008, Gorton et al. 2013, López-Soria et al. 2013, Swoboda-Kopeć et al. 2014), Asia (Fujita et al. 2007, Wahyuningsih et al. 2008, Chowdhary et al. 2010, Sharma et al. 2013, Li et al. 2014, Tay et al. 2014,Feng et al. 2015), and Australia (Lockhart et al. 2009). The total number of*C. nivariensis* isolates described in the literature has been low in these countries. To the best of our knowledge, the isolation of *C. nivariensis* in clinical samples has not been reported to date in countries of North America and South America.

*C. nivariensis* isolates are less susceptible than *C. glabrata*isolates to the azolic antifungal agents [fluconazole (FLC), itraconazole (ITR), and voriconazole (VRC)] that are commonly used in the treatment of candidiasis (Borman et al. 2008). Thus, a periodic monitoring of this species is necessary to determine its antifungal resistance profile (Fujita et al. 2007, Borman et al. 2008).

Virulence factors play a crucial role in the colonisation, adhesion, invasion, dissemination, and escape from host defences. Compared with *C. albicans*, few studies have addressed the expression of virulence factors in*C. glabrata,* including the adhesion of the organism to host cells and/or tissues as well as medical device surfaces, biofilm formation, and the secretion of hydrolytic enzymes (e.g., proteases, lipases, and haemolysins) (Silva et al. 2012)*.* Furthermore, very little is known about the virulence attributes of *C. nivariensis* (Fujita et al. 2007).

Based on the scarce knowledge of this pathogen, the present study aimed to evaluate the antifungal susceptibility profile and the production of virulence attributes in a clinical strain of *C. nivariensis* isolated from a hospital in Rio de Janeiro (RJ), Brazil. In parallel, two reference strains were included with the aim of comparing the evaluated phenotypic markers.

## MATERIALS AND METHODS

*Fungal strains, growth conditions, and biochemical identification* - We analysed a clinical fungal strain obtained from a patient from a public hospital of RJ who was diagnosed with non-Hodgkin’s lymphoma with lesions in the nasal cavities. The clinical strain [893391 strain/Brazilian National Cancer Institute (INCA)] was isolated from a nasal secretion in 2004 and was identified by API 20 C AUX (bioMérieux, France) as *C. glabrata*. This strain was sent to the Mycology Laboratory of the Evandro Chagas National Institute of Infectious Diseases, Oswaldo Cruz Foundation, RJ, for further study. This fungal isolate was grown on Sabouraud dextrose agar and Chromagar *Candida* medium (both at 37ºC for 48 h) to evaluate its viability and purity, respectively. The confirmation of species was achieved by biochemical analysis with the Vitek 2 system (bioMérieux) using a YST card according to the manufacturer’s guidelines. In addition, two reference strains were included, *C. nivariensis* WM 09.150 and *C. glabrata* ATCC 2001, for comparative purposes.

*Molecular identification* - Yeast cells were recovered from Sabouraud dextrose agar and used for DNA extraction with the Gentra^®^Puregene^®^ Yeast and G^+^ Bacteria Kit (Qiagen, Germany). The clinical strain was identified by sequencing the internal transcribed spacer (ITS)1-5.8S-ITS2 region and D1/D2 domains of the 28S of the rDNA as previously described (Alcoba-Flórez et al. 2005b). Sequences were edited using Sequencher^TM^ v.4.9 and compared by BLAST with sequences that were available from the National Center for Biotechnology Information/GenBank database. Phylogenetic analyses were conducted using the MEGA 4.0.2 software (Tamura et al. 2007).

*Antifungal susceptibility testing against planktonic cells* - In vitro antifungal susceptibility testing against planktonic cells was performed according to the recommendations proposed by the Clinical and Laboratory Standards Institute (CLSI) M27-A3 protocol (CLSI 2008a). Amphotericin B (AMB), posaconazole (POS), caspofungin (CAS), FLC, ITR, and VRC (Sigma-Aldrich Chemical Corporation, USA) were tested. Briefly, RPMI-1640 medium with L-glutamine and without bicarbonate (Gibco BRL, Life Technologies, The Netherlands), buffered with 0.165 M 3-*N-*morpholinepropanesulfonic acid (MOPS) at pH 7, was used for the broth microdilution test. The inoculum was prepared from a 24-h fresh Sabouraud dextrose agar culture; the cells were harvested in RPMI medium and diluted to approximately 1-5 × 10^3^ cells mL^-1^. The plates were incubated at 35ºC for 24 h. The minimal inhibitory concentrations of the drugs on planktonic cells (PMICs) were determined according to the CLSI M27-A3/ M27-S3 protocol (CLSI 2008a, b).

*Production of hydrolytic enzymes* - The in vitro production of extracellular hydrolytic enzymes was measured using plate assays (Price et al. 1982). Briefly, protease activity was evaluated using yeast carbon base supplemented with bovine serum albumin (Rüchel et al. 1982), phytase activity was evaluated using the calcium phytate agar plate (Tsang 2011), phospholipase activity was assessed using egg yolk agar plate (Price et al. 1982), esterase activity was determined using the Tween agar plate (Aktas et al. 2002), and haemolytic activity was assayed using the blood agar plate (Luo et al. 2001). In this set of experiments, aliquots (10 µL) of 48-h-old cultured fungal cells (1 × 10^7^cells mL^-1^) were spotted on the surface of the agar medium and incubated at 37ºC for up to seven days. The diameter of the colony (*a*) and the diameter of the colony plus the precipitation zone (*b*) were measured using a digital paquimeter, and the enzymatic activities were expressed as the *Pz* value (*a/b*), as previously described (Price et al*.* 1982). According to this definition, low *Pz*values indicate high enzymatic production and, conversely, high *Pz*values indicate low enzymatic production. The enzymatic activity was scored into four categories: a *Pz* of 1.0 indicated no enzymatic activity, a*Pz* between 0.999-0.700 indicated low enzymatic activity, a*Pz* between 0.699-0.400 indicated moderate enzymatic activity, and a *Pz* between 0.399-0.100 indicated high enzymatic activity (Price et al. 1982).

*Adhesion to abiotic substrates* - The adherent ability to abiotic substrates was tested using glass and polystyrene. The glass slide were first washed with Extran for 2 h and 70% ethanol for 30 min and were then sterilised at 180ºC for 2 h. Fungal cells (1 × 10^6^ cells mL^-1^) were placed on glass slides and on 24-well polystyrene plates and incubated at 37ºC for 1 h. Subsequently, the abiotic substrates were washed three times in phosphate-buffered saline (PBS) to remove nonadherent cells. Five different microscopic fields were counted in each system to express the number of total fungi adhering to these substrates (Reinhart et al. 1985).

*Production and antifungal susceptibility of biofilm-forming cells* - Fungal cell suspensions were adjusted to 1 × 10^3^ cells mL^-1^ in yeast nitrogen base (YNB) medium supplemented with 0.5% glucose and transferred to 96-well polystyrene microtitre plates. The plates were incubated at 37ºC for 48 h to allow biofilm formation. The biomass formation was assessed using crystal violet (CV) staining and the viable cells in biofilm were measured using a colorimetric assay that investigates the metabolic reduction of 2,3-bis (2-methoxy-4-nitro-5-sulfophenyl)-5-[(phenylamino)carbonyl]-2H-tetrazolium hydroxide (XTT) (Sigma-Aldrich) to a water-soluble brown formazan product (Peeters et al. 2008). Briefly, the wells were washed three times in PBS to remove nonadherent cells. An aliquot of 100 µL of 99% methanol was added to each well for 15 min to fix the biofilm, and then the supernatant was discarded. Microplates were air-dried and 200 µL of 0.4% CV solution was added to each well and incubated at room temperature for 20 min. The dye solution was discarded and the wells were washed with 200 µL of sterile distilled water. Finally, 150 µL of 33% acetic acid was added to the stained wells and the absorbance was measured at 590 nm. For the XTT reduction assay, a solution containing 200 µL PBS with 1 mg mL^-1^ XTT (Sigma-Aldrich) and 0.4 mM menadione (Sigma-Aldrich) was used. The plate was incubated in the dark at 37ºC for 3 h. Thereafter, 100 µL of this solution was transferred to another microplate and the colorimetric change was measured at 492 nm using a microplate reader. After biofilm formation, the YNB medium was discarded and the wells were washed with 200 µL of PBS. Then, an aliquot of 100 µL of RPMI-1640 that was buffered with MOPS and supplemented with the antifungals prepared according to the CLSI M27-A3 protocol (CLSI 2008a) was added. The plates were incubated at 37ºC for 24 h. After this last incubation, the CV staining and XTT reduction assay were performed, as described above, to detect cell biomass and viability, respectively. The minimum biofilm eradication concentrations (MBECs) were determined as the lowest concentrations of the antifungal drug that were able to reduce at least 50% of cell biomass or viability compared with the drug free growth control well (Melo et al. 2011).

*Statistical analysis* - All experiments were performed at least twice. The data were analysed statistically in different experimental groups using the Kruskal-Wallis test. To compare data between groups, the Mann-Whitney*U* test was used. p*-*values < 0.05 were considered to be statistically significant. The statistical analyses were performed using the Statistical Package for the Social Sciences v.17.0, for Windows^®^(SPSS Inc, USA).

## RESULTS AND DISCUSSION

Initially, the identification of the clinical strain 893391/INCA was reconfirmed to certify its authenticity by mycology methodologies. The cultivation in chromogenic CHROMagar *Candida* medium generated white colonies with a smooth texture. Subsequently, both the carbohydrate assimilation and the metabolic enzymatic profiles were evaluated using the Vitek 2 system, which identified the clinical strain 893391/INCA as *C. glabrata* (98% probability). However, phenotypic tests were not able to discriminate among the three species of the *C. glabrata* complex; therefore, molecular methods were applied to confirm the identification of this clinical strain (Alcoba-Flórez et al. 2005b, Wahyuningsih et al. 2008, Romeo et al. 2009, Enache-Angoulvant et al. 2011, López-Soria et al. 2013).

Genomic sequences obtained from our clinical strain showed 100% similarity with the GU199444 (ITS region) and AF313362 (D1/D2 domains) sequences found in the GenBank database, thus confirming its identity as *C. nivariensis* rather than *C. glabrata* (Figure). The obtained sequences with respect to the ITS1-5.8S-ITS2 region and D1/D2 domains of the 28S of the rDNA of the 893391/INCA strain were deposited in GenBank under the accessions KJ957824 and KJ957825, respectively.

**Figure:**
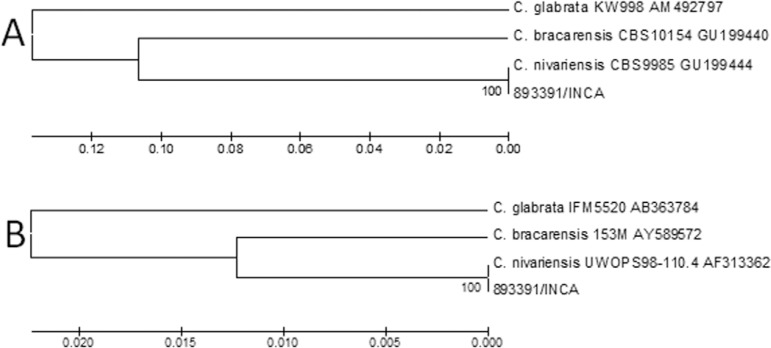
Dendrograms based on analysis of internal transcribed spacer (ITS) (A) and D1/D2 (B) regions of the 893391 strain and sequences from GenBank. The evolutionary histories of both trees were inferred using the UPGMA method. The percentage of replicate trees in with the associated taxon clustered in the bootstrap test (1,000 replicates) is show next to the branches. The evolutionary distances were computed using the maximum composite likelihood method and were in expressed as the number of base substitutions per site. There were 700 ITS and 580 D1/D2 positions in the final dataset.

According to a simple review of the current published literature (Alcoba-Flórez et al. 2005a, Fujita et al. 2007, Borman et al. 2008, Wahyuningsih et al. 2008, Lockhart et al. 2009, Chowdhary et al. 2010, Gorton et al. 2013, López-Soria et al. 2013, Sharma et al. 2013, Li et al. 2014, Swoboda-Kopeć et al. 2014,Tay et al. 2014, Feng et al. 2015), 55 isolates of *C. nivariensis* have been described in 35 cases in European countries, 19 cases in Asia, and one case report in Australia (Table I). Therefore, there are no cases of *C. nivariensis* in the American continent. Hence, to the best of our knowledge, this is the first report of *C. nivariensis* in South America, specifically in Brazil. *C. nivariensis* has been found in different clinical samples, including bronchoalveolar lavage, blood, urine, catheter, sputum, lung biopsy, pleural fluid, vaginal swab, toenail, and other clinical specimens, but until now, it has not been isolated from a nasal secretion as the 893391/INCA strain (Table I).

**TABLE I:** *Candida nivariensis* isolates described in the literature

Reference	Country	Total of isolates (n)	Source of isolates (n)	Clinical details	Susceptibility antifungal profile		

					S/low MIC	SDD/I	R/high MIC
Alcoba-Flórez et al. (2005b)	Spain	3	Broncho-alveolar lavage (1), blood culture (1), urine (1)	Multiple pulmonary abscesses, pancreatic abscess, lumbar pain	Not stated		
Fujita et al. (2007)	Japan	1	Catheter (1)	Rheumatoid arthritis	-	-	FLC
Wahyuningsih et al. (2008)	Indonesia	1	Oral rinse (1)	HIV infection	FLC, ITC, VRC and CAS/AMB, 5FC, POS, and ISA	-	-
Borman et al. (2008)	UK	16	Blood (3), oral cavity (3), pelvic abscess (2), ascetic fluid (1), peritoneal fluid (1), lung biopsy (1), not stated (5)	Oral candidosis, neutropaenia, pneumonia, malignancy, peritonitis, and others	-	-	FLC and ITC/5FC and VRC
Lockhart et al. (2009)	Australia	1	Pleural fluid (1)	Not stated	FLC	-	-
Chowdhary et al. (2010)	India	2	Sputum (1), blood (1)	HIV infection, diabetes	FLC, ITC, VRC and CAS/AMB, 5FC, and POS	-	-
López-Soria et al. (2013)	Spain	2	Blood (1), catheter (1)	Intestinal fistula associated to a severe malnutrition	Echinocandins, FLC, and VRC/AMB	ITC	-
Sharma et al. (2013)	India	5	High vaginal swab (4), bronchoalveolar lavage (1)	Vulvovaginal candidiasis, not stated	VRC, ITC and echinocandins/AMB, POS, and ISA	-	FLC
Gorton et al. (2013)	UK	1	Urine (1)	Renal transplant	-	-	FLC
Li et al. (2014)	China	7	Vaginal swab (7)	Vulvovaginal candidiasis	NYT	FLC, ITC, MCN, and CLT	-
Tay et al. (2014)	Malaysia	2	Blood culture (1), vaginal swab (1)	Not stated, vulvovaginal candidiasis	FLC, VRC, and CAS/AMB and POS	-	CLT
Swoboda-Kopeć et al. (2014)	Poland	13	Urine (8), lower respiratory tract (3), surgical specimens (1), body fluids (1)	Not stated	Not stated		
Feng et al. (2015)	China	1	Toenail (1)	Not stated	FLC, VRC, and POS	-	ITC
Present paper (2015)	Brazil	1	Nasal secretion (1)	Non-Hodgkin lymphoma	VRC and CAS/AMB	ITC	FLC/POS

AMB: anfotericin B; CAS: caspofungin; CLT: clotrimazole; FLC: fluconazole; HIV: human immunodeficiency virus; I: intermediate; ISA: isaconazole; ITC: itraconazole; MCN: iconazole; MIC: minimal inhibitory concentration; NYT: nistatina; POS: posaconazole; R: resistant; S: susceptible; SDD: susceptible-dose dependent; VRC: voriconazole; 5FC: flucytosine.

Concerning the antifungal susceptibility profile (Table II), both the *C. nivariensis* 893391/INCA strain and the reference strains presented low PMICs to AMB, CAS, and VRC. The low MICs to AMB, CAS, and VRC reported herein corroborate the results of previous studies (Chowdhary et al. 2010, Sharma et al. 2013, Gil-Alonso et al. 2015). However, only our strain showed elevated PMICs for POS and ITC as well as FLC resistance. In a study conducted by Borman et al. (2008), 16 isolates of *C. nivariensis*were tested for seven antifungals, including POS and ITC. These isolates showed high PMIC values (2 and > 16 mg L^-1^, respectively) to these drugs. Similar results were found in the present study. Other studies also revealed high FLC PMICs (Fujita et al. 2007, Borman et al. 2008, Sharma et al. 2013) (64, 128, 16 mg L^-1^, respectively). Despite the lack of clinical breakpoints (CBP) and defined epidemiological cut-off values (ECV) for this species, *C. nivariensis*has been described in the literature as an emerging pathogenic fungus with a varying susceptibility to azoles (Borman et al. 2008). According to the CBP and ECV of its sibling species *C. glabrata* (Pfaller & Diekema 2012, Espinel-Ingroff et al. 2014), our strain would be resistant to FLC (PMIC ≥ 64 mg L^-1^) and non-wild type to FLC (ECV > 8 mg L^-1^) and POS (ECV > 1 mg L^-1^) (Table II).

**TABLE II:** Antifungal susceptibility of *Candida nivariensis* and*Candida glabrata* planktonic and sessile cells

Antifungals	PMIC (mg L^-1^)/susceptibility profile^*a*^			CV MBEC (mg L^-1^)/% reduction of biomass			XTT MBEC (mg L^-1^)/% reduction of viability		
		
	*C. nivariensis*		*C. glabrata*	*C. nivariensis*		*C. glabrata*	*C. nivariensis*		*C. glabrata*
					
	893391/INCA	WM 09.150	ATCC 2001	893391/INCA	WM 09.150	ATCC 2001	893391/INCA	WM 09.150	ATCC 2001
AMB	0.25/NA^*b*^	0.25/NA^*b*^	0.12/NA^*b*^	≥ 16/54.2	≥ 16/30.7	≥ 16/28.4	≥ 16/54	≥ 16/73.1	≥ 16/72.8
CAS	0.03/S	0.12/S	0.12/S	≥ 8/31.6	≥ 8/22.8	≥ 8/40.4	≥ 8/63.7	≥ 8/0	≥ 8/79.8
FLC	≥ 64/R	8/SDD	8/SDD	≥ 64/6.7	≥ 64/23.7	≥ 64/13.7	≥ 64/1.5	≥ 64/15	≥ 64/0
ITC	0.25/SDD	0.06/S	0.06/S	≥ 16/19.1	≥ 16/31.1	≥ 16/1.7	≥ 16/16	≥ 16/0	≥ 16/10.7
VRC	0.25/S	0.03/S	0.03/S	≥ 16/13.1	≥ 16/41.7	≥ 16/20.5	≥ 16/20	≥ 16/7	≥ 16/22
POS	2/NA^*c*^	0.06/NA^*c*^	0.03/NA^*c*^	≥ 16/21.5	≥ 16/24.3	≥ 16/6.9	≥ 16/54.5	≥ 16/29.3	≥ 16/13.8

*a*: determined according to Clinical and Laboratory Standards Institute (CLSI) M27-S3 (CLSI 2008b); *b*: the CLSI has not defined interpretative cut-offs for amphotericin B (AMB). In general, isolates with AMB minimal inhibitory concentration (MIC) >1 mg L^-1^ are likely to be resistant to this drug (CLSI 2008a); *c*: breakpoints not established by CLSI (CLSI 2008b, 2012); CAS: caspofungin; CLT: clotrimazole; CV: crystal violet; FLC: fluconazole; ITC: itraconazole; MBEC: minimum biofilm eradication concentration; NA: not applicable; PMIC: MIC of the drugs on planktonic cells; POS: posaconazole; R: resistant; S: susceptible; SDD: susceptible-dose dependent; VRC: voriconazole; XTT: 2,3-bis (2-methoxy-4-nitro-5-sulfophenyl)-5-[(phenylamino)carbonyl]-2H-tetrazolium hydroxide.

The pathogenicity of *Candida* species is associated with a multitude of virulence factors, including the ability to evade host defences, adhesion to host tissue and/or medical devices, the ability to form biofilm and the production of tissue-damaging hydrolytic enzymes such as proteases, lipases, and haemolysins (Silva et al. 2012). Along this line of thinking, we demonstrated that the *C. nivariensis* 893391/INCA strain, the *C. nivariensis* WM 09.150 reference strain, and the*C. glabrata* ATCC 2001 type strain were excellent protease producers (Table III), with no significant differences in the *Pz* values of these strains (p > 0.05). Jang et al*.* (2011) also found protease activity in six of 38 *C. glabrata* isolates from fresh feral pigeon faeces, but the production of proteases was moderate (mean *Pz* = 0.65 ± 0.17). Because *C. glabrata* does not possess classical secreted aspartic protease genes in its genome (Parra-Ortega et al. 2009, Silva et al. 2014), we believe that the enzymatic degradation of albumin verified herein may be due the production of yapsins (YPS). YPS are a family of five nonsecreted glycosylphosphatidyinositol (GPI)-linked aspartic proteases in *Saccharomyces cerevisiae* that have homologues in *C. glabrata*, which have a well-known role in cell wall integrity (Krysan et al. 2005) and cell-cell interactions (Silva et al. 2014). The role of YPS-family proteases coded by 11 genes is known among the *C. glabrata* virulence factors that have been described. The expression of YPS genes significantly increases the capacity of the fungus to survive inside human macrophages (Krysan et al. 2005) *.* Swoboda-Kopeć et al. (2014) confirmed the prevalence of three genes (*YPS2*, *YPS4*, and*YPS6*) in the majority of *C. glabrata* strains isolated from clinical specimens. However, the prevalence of these genes in 13 clinical isolates of *C. nivariensis* was low (Swoboda-Kopeć et al. 2014). Regarding the phytase activity,*C. nivariensis* 893391/INCA strain and *C. nivariensis* WM 09.150 reference strain presented a moderate production of phytase, whereas the type strain of *C. glabrata* exhibited a low production of this enzyme (Table III). Phytase is a phosphohydrolase that cleaves phytate and releases inorganic phosphate and inositol, which are both essential nutrients for all living cells (Lei & Porres 2003). In *Candida*species, such as the *Candida parapsilosis* complex (Abi-Chacra et al. 2013), maintaining a supply of inositol and phosphate mediated by phytase seems to be especially important for pathogen survival and persistence in the host (Tsang 2011). In this study, the three tested strains were negative for the production of phospholipase, esterase, and haemolysins under the employed experimental conditions. Udayalaxmi et al. (2014) did not find phospholipase activity in 14 *C. glabrata*strains isolated from the genitourinary tract, but all of their *C. glabrata* strains presented haemolytic activity.

**TABLE III:** Production of hydrolytic enzymes, adhesion to abiotic substrates, and biofilm formation detected in *Candida nivariensis* and*Candida glabrata* strains

Species/code	Hydrolytic activities		Adhesion to abiotic substrates (1 h)		Biofilm	
		
	Protease (*P*_*z*_)^*a*^	Phytase (*P*_*z*_)^*a*^	Glass^*b*^	Polystyrene^*b*^	Biomass^*c*^	Viability^*c*^
*C. nivariensis*						
893391/INCA	0.347 ± 0.040	0.629 ± 0.025	17.0 ± 4.4	25.4 ± 4.9	0.330 ± 0.045	1.173 ± 0.054
WM 09.150	0.375 ± 0.000	0.634 ± 0.047	14.0 ± 3.0	23.9 ± 2.4	0.440 ± 0.095	0.811 ± 0.080
*C. glabrata*						
ATCC 2001	0.357 ± 0.034	0.714 ± 0.00	16.0 ± 3.0	24.1 ± 5.3	0.858 ± 0.009	1.084 ± 0.028

*a*: the protease and phytase activities were measured by the formation of a clear halo around the colony and expressed as*Pz* value as previously described (Price et al. 1982) (the*Pz* value was scored into four categories:*Pz* of 1.0 indicated no enzymatic activity,*Pz* between 0.999-0.700 indicated low enzymatic activity, *Pz* between 0.699-0.400 corresponded to moderate enzymatic activity, and *Pz* between 0.399-0.100 mean high enzymatic activity); *b*: the results were expressed as number of fungal cells per microscopic field; *c*: the biomass and viability of biofilm were measured by crystal violet incorporation at 540 nn and 2,3-bis (2-methoxy-4-nitro-5-sulfophenyl)-5-[(phenylamino)carbonyl]-2H-tetrazolium hydroxide reduction at 492 nm, respectively. All the results were reported as the arithmetic means ± standard deviation.

*C. nivariensis* 893391/INCA strain, as well as both reference strains, was able to bind to inert surfaces, with a predilection to polystyrene compared to glass (p < 0.001) (Table III). Adhesion is a crucial step for beginning and establishing an infectious process. The adhesive ability of *Candida* species is associated with the presence of specific cell-wall glycoproteins known as adhesins. The ability of *C. glabrata* to adhere to host epithelial tissue is mediated by a number of GPI-linked adhesion genes, including the *EPA* gene family (De Groot et al. 2008). In *C. glabrata,*the deletion of the *EPA1* gene reduces the in vitro adhesion to epithelial cells, thus highlighting the essential role of this gene in adherence to biotic substrates (Cormack et al. 1999, d, de Las Peñas et al. 2003). In contrast, *EPA6*-mediated adhesion is engaged in strong hydrophobic interactions with abiotic surfaces and is the principal adhesin involved in biofilm formation (El-Kirat-Chatel et al. 2015).

Biofilm formation is considered to be an important virulence attribute of*Candida* species and is associated with recurrent infections and treatment failures by limiting the penetration of drugs through the biofilm matrix (Mukherjee & Chandra 2004). In this study, *C. glabrata* ATCC 2001 type strain produced a significantly greater amount of biofilm biomass (p < 0.001) than did both *C. nivariensis* 893391/INCA and *C. nivariensis* WM 09.150 reference strain (Table III). The viability of cells forming biofilm showed a significant difference among *C. nivariensis* WM 09.150 and *C. glabrata* ATCC 2001 type strain (p < 0.05) as well as between 893391/INCA strain and WM 09.150 (p < 0.05) (Table III). Our strain, however, showed a biofilm profile of viability, as determined by the XTT assay, which was more related to the *C. glabrata* ATCC 2001 type strain (p > 0.05) (Table III). According to the literature,*C. glabrata* clinical isolates are capable of forming biofilm and their presence during the infection has been associated with higher morbidity and mortality rates compared with isolates that are unable to form biofilm (Kumamoto 2002).

To determine whether antifungal agents (AMB, CAS, FLC, ITC, VRC, and POS) could disarticulate the biofilms, they were exposed to different concentrations of antifungal agents. No significant reductions in the number of viable cells were observed for the lower concentrations of antifungal agents tested against the three analysed strains (data not shown). In the present study, the highest tested concentration of AMB, CAS, and POS (16, 8, and 16 mg L^-1^, respectively) was able to inhibit the viability of *C. nivariensis* 893391/INCA strain by more than 50% compared with the nontreated fungal cells. Similar results with these antifungal drugs were found for the *C. nivariensis* WM 09.150 reference strain and *C. glabrata* ATCC 2001 type strain. The reduction in viability for FLC, ITC, and VRC was low, at less than 30% for each of the strains. Concerning the total biomass (Table II), *C. nivariensis* 893391/INCA strain also presented a greater than 50% reduction of biomass at an AMB concentration of 16 mg/L. The highest concentrations of the other antifungal drugs yielded less than a 32% reduction in biomass for our clinical strain. The two reference strains presented less than a 42% reduction in biomass for all of the highest concentrations of the six tested antifungal drugs.

We observed that our MBECs values are much higher than PMICs values for all six tested antifungal drugs. Similar results were found for *C. albicans*, *Candida tropicalis*, *Candida parapsilosis*, *Candida orthopsilosis*, and*Candida metapsilosis* (Melo et al. 2011). Fonseca et al. (2014)evaluated the effects of FLC on the formation and control of *C. glabrata*biofilm and they did not observe a reduction in the number of viable cells, even when antifungal drugs were applied at high concentrations. Therefore, further studies utilising higher antifungal concentrations are necessary to better determine the MBECs values of *C. glabrata* and *C. nivariensis*strains.

The present study describes, for the first time, the isolation of *C. nivariensis* from an oncologic patient in Brazil and reports its potential antifungal resistance to FLC, which is the most common antifungal drug used for candidiasis treatment. As a warning, other countries in the Americas need to search for *C. nivariensis* by means of molecular methods in their*C. glabrata* culture collections because standard biochemical analytical methods are not sufficient to properly identify *C. niva- riensis.*We strongly believe that the real incidence of *C. nivariensis*in our continent may be underestimated due to the lack of adequate molecular surveillance strategies.

In addition, to our knowledge, this is the first study to evaluate the in vitro production of extracellular hydrolytic enzymes and biofilm formation ability in clinical strain of *C. nivariensis.* Further studies are needed to monitor the frequency of this species in clinical isolates, their potential virulence factors, and their susceptibility to antifungals, mainly due to the phenomenon of azolic resistance.
